# The role of the nucleus basalis of Meynert in neuromodulation therapy: a systematic review from the perspective of neural network oscillations

**DOI:** 10.3389/fnagi.2024.1376764

**Published:** 2024-04-08

**Authors:** Liwu Jiao, Huicong Kang, Yumei Geng, Xuyang Liu, Mengying Wang, Kai Shu

**Affiliations:** ^1^Department of Neurosurgery, Tongji Hospital, Tongji Medical College, Huazhong University of Science and Technology, Wuhan, Hubei, China; ^2^Department of Neurology, Tongji Hospital, Tongji Medical College, Huazhong University of Science and Technology, Wuhan, Hubei, China

**Keywords:** nucleus basalis of Meynert, neurophysiology, Alzheimer’s disease, deep brain stimulation, neural network, oscillations, cross-frequency coupling

## Abstract

As a crucial component of the cerebral cholinergic system and the Papez circuit in the basal forebrain, dysfunction of the nucleus basalis of Meynert (NBM) is associated with various neurodegenerative disorders. However, no drugs, including existing cholinesterase inhibitors, have been shown to reverse this dysfunction. Due to advancements in neuromodulation technology, researchers are exploring the use of deep brain stimulation (DBS) therapy targeting the NBM (NBM-DBS) to treat mental and neurological disorders as well as the related mechanisms. Herein, we provided an update on the research progress on cognition-related neural network oscillations and complex anatomical and projective relationships between the NBM and other cognitive structures and circuits. Furthermore, we reviewed previous animal studies of NBM lesions, NBM-DBS models, and clinical case studies to summarize the important functions of the NBM in neuromodulation. In addition to elucidating the mechanism of the NBM neural network, future research should focus on to other types of neurons in the NBM, despite the fact that cholinergic neurons are still the key target for cell type-specific activation by DBS.

## 1 Introduction

As the largest group of cholinergic neurons in the basal forebrain, the nucleus basalis of Meynert (NBM) is a crucial source of cholinergic efferents to the neocortex and plays an indispensable role in supporting vital brain functions, such as arousal, attention, multimodal encoding, visual processing, and experience-dependent cortical plasticity (Goard and Dan, 2009; Sanchez-Alavez et al., 2014; Liu et al., 2015; Ljubojevic et al., 2018; Martinez-Rubio et al., 2018; Meir et al., 2018; Wan et al., 2019). NBM dysfunction is implicated in multiple neuropsychiatric and neurological disorders, including Alzheimer’s disease (AD), schizophrenia, Parkinson’s disease (PD), Lewy body dementia (LBD), and Down syndrome (Williams et al., 2013; González et al., 2021; Mehraram et al., 2022; Schumacher et al., 2022). The involvement of the NBM may be a critical prodrome or an early molecular cascade event. For instance, research has demonstrated that in AD, the earliest accumulation of neurofibrillary tangles (NFTs) occurs in the NBM, preceding the accumulation in the entorhinal cortex and locus coeruleus (Braak et al., 2011; Arendt et al., 2015; Chung et al., 2016; Hanna et al., 2020). Drugs that regulate the activity of cholinergic neurons in the NBM and its cortical efferent have been proposed as promising therapeutic approaches for various neurodegenerative diseases. However, current drugs targeting this pathway, including existing cholinesterase inhibitors, have limited efficacy and cannot halt or reverse neurodegenerative diseases (Yu et al., 2021). Based on advancements in neuromodulation technology, deep brain stimulation (DBS) has been proven to be the most effective strategy for treating neurodegenerative diseases due to its precise circuit-targeted neuromodulation (Krauss et al., 2021). Therefore, DBS has been approved by the Food and Drug Administration (FDA) for treating PD, essential tremor, dystonia and other movement disorders, and it has yielded satisfactory clinical outcomes and evidence (Krauss et al., 2021). In addition, DBS has been used to treat pain syndromes, such as neuropathic pain and cluster headache, as well as epilepsy (Pereira and Aziz, 2014; Lee et al., 2019). Based on the key role of the NBM in cognition, many published studies have focused on the effect of cognitive neuromodulation via DBS targeting the NBM (NBM-DBS) in various diseases (Kuhn et al., 2015; Gratwicke et al., 2018, 2020; Cappon et al., 2022; Sasikumar et al., 2022). A phase I clinical trial conducted by Kuhn et al. (2015) investigated the effects of NBM-DBS on the treatment of AD, revealing a mere 3-point deceleration in the rate of progression according to the Alzheimer’s Disease Assessment Scale-Cognitive Subscale (ADAS-Cog) and an increase in cerebral cortical glucose metabolism revealed by [18F]-fluoro-desoxyglucose-PET at a 12-month follow-up. Davide Cappon et al. explored the effects of NBM-DBS in five Parkinson’s disease dementia (PDD) patients, two of whom experienced slower cognitive decline; however, the Mini-Mental State Examination (MMSE) score still exhibited an annual mean decrease of 0.3 points at the 36-month follow-up (Cappon et al., 2022). However, studies from Sasikumar S and Gratwicke J found that NBM-DBS did not improve cognitive function in PDD patients (Gratwicke et al., 2018; Sasikumar et al., 2022). In addition, Gratwicke J found that although no consistent improvements were observed in exploratory clinical outcome measures for NBM-DBS, but the severity of neuropsychiatric symptoms reduced with NBM-DBS in 3 of 5 dementia with Lewy bodies (DLB) patients (Gratwicke et al., 2018).

The mechanisms of NBM-DBS have also been investigated recently in animal models of AD, and a variety of hypotheses have been proposed, such as regulating the cholinergic system (Hotta et al., 2009), modulating regional glucose metabolism (Wang et al., 2018), increasing regional cerebral blood flow (Adachi et al., 1990a,b), providing neuroprotective effects (Temel et al., 2006; Wallace et al., 2007) and regulating neural circuits (Singh et al., 2022). However, the significant treatment effects of NBM-DBS on cognitive dysfunction that have been observed in animal studies have not been replicated in clinical trials. Therefore, NBM-DBS is still in the preliminary stage of exploration.

The current review aims to provide a comprehensive update and summary of progress on cognition-related neural networks or circuits as well as the neuroanatomical and neurophysiological properties of the NBM. Additionally, this study aims to provide evidence from animal and clinical studies regarding the effect of NBM-DBS on cognitive dysfunction in various neurological diseases. The findings of this study will provide a basis for obtaining a precise understanding of the important and promising role of the NBM in neuromodulation.

## 2 Neural network oscillations related to cognition

Rhythmic neural electrical activity, also known as oscillations, has attracted research attention for nearly a century. Behavior-dependent oscillations are the result of dynamic interactions between intrinsic cellular and circuit properties within the brain and are ubiquitous in mammals (Berger, 1929; Berger, 1969; Llinas, 1988; Steriade, 2001; Laurent, 2002; Destexhe and Sejnowski, 2003). The neural network contains multiple frequency bands of oscillations, ranging from 0.05 Hz to 500 Hz, which are categorized as slow oscillations (<1.5 Hz), δ oscillations (1.5–4 Hz), θ oscillations (4–8 Hz), α oscillations (8–13 Hz), β oscillations (13–30 Hz), γ oscillations (30–80 Hz) and fast oscillations (>100 Hz) based on frequency (Jasper and Penfield, 1949; Nokia and Penttonen, 2022). Oscillations are currently thought to be shaped by the summations of thousands of neurons in a particular brain region (Engel et al., 2001; Varela et al., 2001). In normal cognitive processes, each oscillatory frequency band is associated with a specific behavioral or executive function. Studies have shown that the δ oscillation phase correlates with the reaction time of behavior and plays a role in neural group synchronization in multiple brain regions (Stefanics et al., 2010). In addition, these oscillations are often associated with resting states, and slow oscillations (<1 Hz) can trigger thalamically generated spindles (Amzica and Steriade, 1998). θ oscillations are believed to play a crucial role in almost all cognitive functions. Previous studies have revealed the key roles of these proteins in learning, memory and synaptic plasticity (Herweg et al., 2020; Senoussi et al., 2022). Additionally, it is common for pyramidal neurons to fire phase locking with θ oscillations, indicating their key roles in interconnecting various brain regions (Pare and Gaudreau, 1996; Buzsaki, 2002; Jacobs et al., 2007; Rutishauser et al., 2010). θ oscillations are also the fundamental bands for cross-frequency coupling (CFC), as they synchronizing with the gamma band in a phenomenon known as θ–γ cross-frequency coupling; the role of θ–γ cross-frequency coupling in working memory has been extensively studied (Lisman and Jensen, 2013). α oscillations are also the most widely understood frequency bands, mostly due to their well-defined roles in attention needs and visual perception (Başar et al., 1997; Klimesch, 2012). β oscillations are often associated with motor-induced events (Pittman-Polletta et al., 2018). Synchronization of β oscillations has always been an indicator of normal motor system function and has also been shown to play a role in working memory (Schmidt et al., 2019). Finally, γ oscillations also play a crucial role in memory, as observed in the θ-γ coupled oscillations throughout the hippocampus and cortex (Lisman and Buzsaki, 2008).

**Figure 1:**
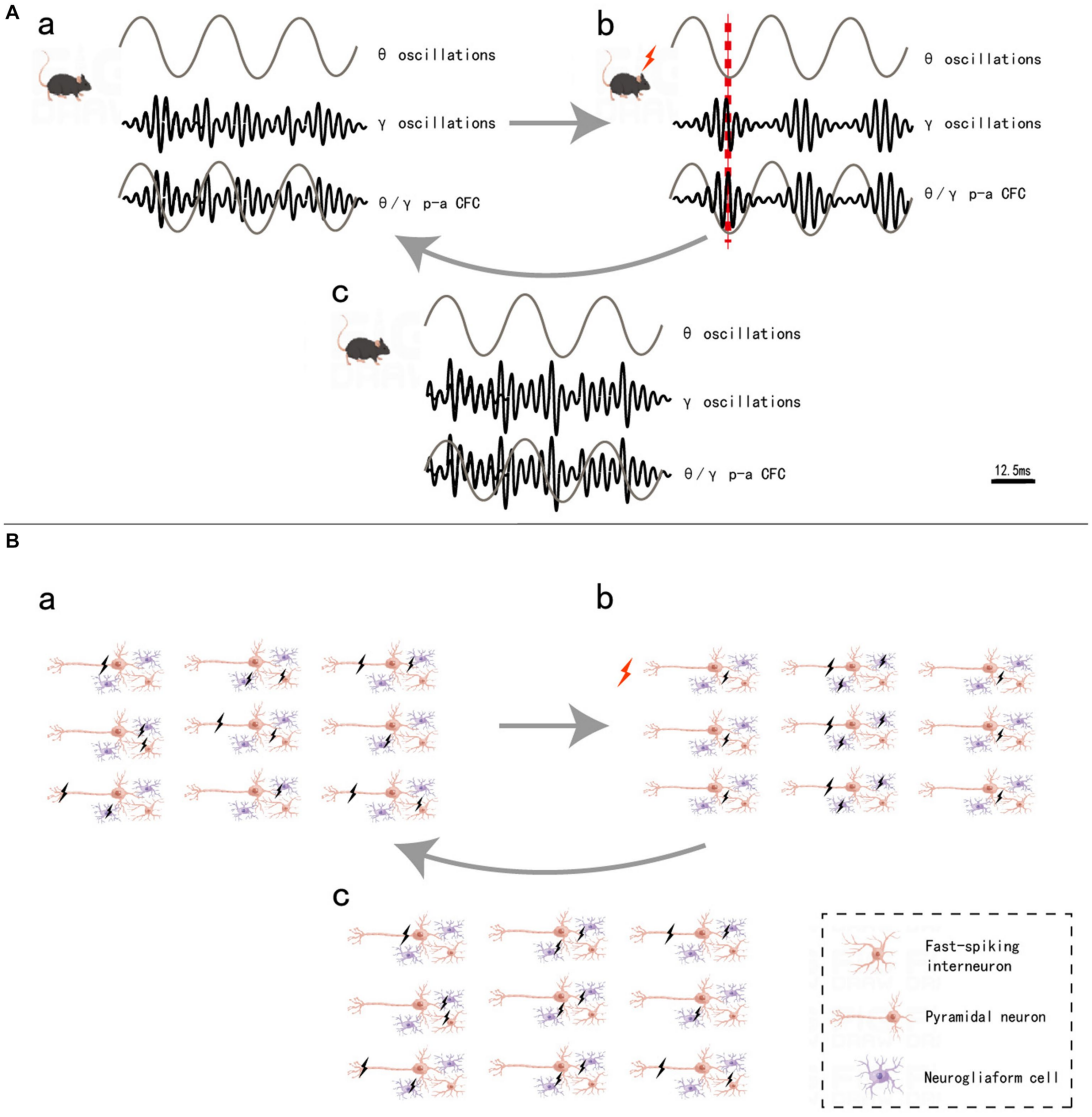
θ/γ p-a CFC regulation **(A)** and neuronal firing pattern **(B)**. a, in mice without stimulation, θ oscillations did not form coupling with γ oscillations **(A)**, and the firing of the three types of neurons was irregular **(B)**. b, when mice are stimulated, the fast-spiking interneurons synchronous firing (IPSP) causes γ oscillations, θ oscillations and γ oscillations, thus leading to θ-γ p-a CFC **(A)**. The pyramidal neurons, which are inhibited by GABA released by interneurons, do not fire, while the uninhibited pyramidal neurons form an ensemble and fire synchronously, completing the information encoding. Additionally, neurogliaform cells that form synaptic connections with this pyramidal neuronal ensemble also form delayed synchronous firing **(B)**. c, after a very short interval when the information processing is done, the delayed synchronous firing from neurogliaform cells cause the release of GABA from the synaptic, which inhibits the pyramidal neuronal ensemble and stops fire **(B)**, and decouples of the θ-γ p-a CFC and return to state awaiting to receive new information **(A)**. Black lightning bolt, neuronal firing, red lightning bolt and red dashed line, the moment when mice are stimulated.

Although each frequency band appears to be separated into a variety of behavioral or cognitive functions, synchronization between different bands through CFC analysis has provided new insights into the complexity of neural networks (Palva and Palva, 2007). CFCs are thought to be integral to the spatiotemporal activation of specific cortical circuits (Canolty and Knight et al., 2010; Yakubov et al., 2022). For example, phenomena such as the θ-γ phase-amplitude (p-a) CFC – which refers to the γ amplitude modulated by the θ phase (Bragin et al., 1995; Montgomery et al., 2008; Colgin et al., 2009; Sakalar et al., 2022) and is known for its crucial role in working memory and memory formation in the hippocampal CA1 region – are among the many modes of CFCs (Lisman and Jensen, 2013). By optogenetic selectively targeting fast-spiking GABAergic interneurons in the barrel cortex *in vivo*, Cardin JA et al. tested whether the synchronous activity of fast-spiking GABAergic interneurons generates γ oscillations, which produce rhythmic inhibitory postsynaptic potentials (IPSPs) to distal dendrites of local pyramidal neurons; their findings showed that the rhythmic inhibition produced excitatory neural ensemble synchrony to process information (Cardin et al., 2009). Sakalar E et al. found that neurogliaform cells (NGFCs), which form synaptic connections with excitatory neuronal ensembles, lead to delayed synchronous firing; moreover, this delay leads to GABAergic inhibition to excitatory neural ensembles when information processing is complete, thereby resulting in actively disengaged excitatory neural ensemble synchronization that enables the formation of new patterns of information processing (Sakalar et al., 2022). These findings represent compelling examples of the significance of cell type-specific activation in rhythmic neural electrical activity and provide a reference for research on the mechanism underlying neural networks. Based on these studies, we constructed a schematic diagram of how θ-γ p-a CFC regulates neuronal firing patterns and cognitive processes (Figure 1).

The cholinergic system plays a key role in controlling neural oscillations throughout the brain and cortical structures (Semba and Fibiger, 1989). Cholinergic neurons suppressed specific low-frequency oscillations, including δ, θ, and α oscillations, while high-frequency oscillations, such as β and γ oscillations, were accompanied by increased acetylcholine release in the thalamus and cortex (Steriade, 2004). Furthermore, cholinergic neurons appear to play a crucial role in influencing θ oscillations in the hippocampus. Acetylcholine (ACh) levels are directly related to θ oscillations in hippocampal neurons and have a direct effect on the amplitude of θ waves (Danışman et al., 2022). θ-γ p-a CFC also appears to rely heavily on ACh modulation, with detected cues evoking phasic ACh release as well as neuronal synchrony across several frequency bands and the emergence of θ-γ coupling (Howe et al., 2017). It is reasonable to assume that the NBM, as the largest group of cholinergic neurons in the basal forebrain, plays an independent role in the regulation of nerve concussion. Although the NBM acetylcholine system has an important influence on neural network oscillations, as shown by Rodriguez R et al., the cholinergic system may also regulate neural network oscillations through muscarinic receptors in addition to nicotinic receptors (Rodriguez et al., 2004), indicating that other types of NBM neurons or receptor components also play an important role in the generation and regulation of neural network oscillations. There are few relevant studies, which need to be explored in the future.

## 3 Anatomy and electrophysiology properties of the NBM

### 3.1 Anatomy of the NBM

**Figure 2:**
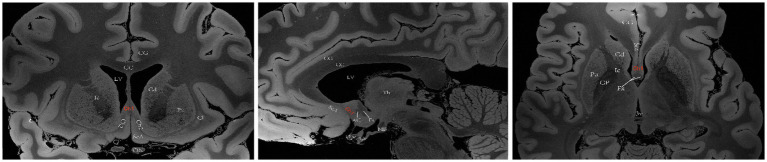
A diagram depicting the location of the human Ch1 on MRI. Images sourced from https://openneuro.org/datasets/ds002179/versions/1.1.0 (Marceglia et al., 2021). Ch1 represents the cholinergic component of MS. Coronal (left), sagittal (middle), axial (right). LV lateral ventricle, CG cingulate gyrus, 3 V third ventricle, Fx Fornix, Th thalamus, Cd caudatum, Cl claustrum, Gp globus pallidus, Pu putamen, Ic Internal capsule, ON optic nerve, Opc optic chiasma, CC corpus callosum, ScA subcallosal area, AC anterior commissure, MB mamillary body, Cy cyathus.

**Figure 3:**
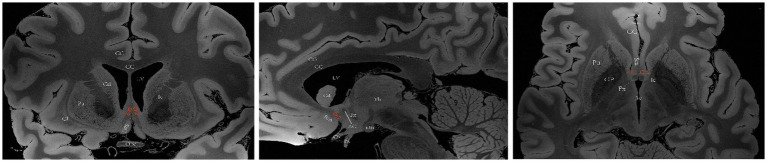
A diagram depicting the location of the human Ch2 on MRI. Images sourced from https://openneuro.org/datasets/ds002179/versions/1.1.0. (Marceglia et al., 2021) Ch2 corresponds to the cholinergic component of the vertical branch of DBB. Coronal (left), sagittal (middle), axial (right). LV lateral ventricle, CG cingulate gyrus, 3 V third ventricle, Fx Fornix, Th thalamus, Cd caudatum, Cl claustrum, Gp globus pallidus, Pu putamen, Ic Internal capsule, Opc optic chiasma, CC corpus callosum, ScA subcallosal area, AC anterior commissure, MB mamillary body, Ps pituitary stalk.

**Figure 4:**
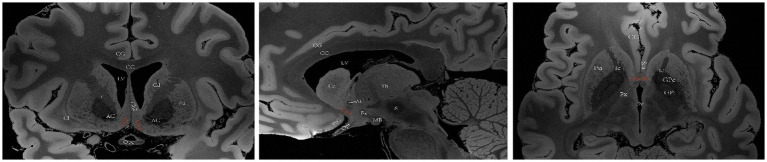
A diagram depicting the location of the human Ch3 on MRI. Images sourced from https://openneuro.org/datasets/ds002179/versions/1.1.0.33 (Marceglia et al., 2021). Ch3 refers to the cholinergic component of the horizontal branch of DBB. Coronal (left), sagittal (middle), axial (right). LV lateral ventricle, CG cingulate gyrus, 3 V third ventricle, Fx Fornix, Th thalamus, Cd caudatum, Cl claustrum, GPi internal globus pallidus, GPe external globus pallidus, Pu putamen, Ic Internal capsule, Opt optic tract, Opc optic chiasma, PtG paraterminal gyrus, CC corpus callosum, ScA subcallosal area, AC anterior commissure, MB mamillary body, R red nucleus.

The basal forebrain is one of the four regions containing cholinergic neurons in the mammalian brain (the others being the brainstem, striatum, and limbic system). This region is anatomically situated above the optic nerve, below the anterior commissure and medially adjacent to the lateral ventricular wall, and it includes the medial septal nucleus (MS), the diagonal band of the Broca nucleus (DBB) (with vertical and horizontal branches), the preoptic nucleus, the basal ganglia (NB) and the anonymous substance (SI) (Woolf, 1991). Mesulam et al. investigated acetyl cholinergic neurons in nonhuman primate brain tissue and introduced the Ch1-Ch4 nomenclature to classify four distinct cholinergic neuron groups located in the basal forebrain. Ch1 corresponds to the MS; many of its neurons are embedded among the fibers of the precommissural fornix, and approximately 10% of its neurons are cholinergic, thus providing a substantial projection to the hippocampus (Figure 2). Ch2 corresponds to the vertical limb of DBB; at least 70% of its neurons are cholinergic, and they merge with the Ch1 cell group dorsally and with Ch3 and Ch4 ventrally. Furthermore, Ch2 is the major source of innervation that the hippocampus and hypothalamus receive from the basal forebrain (Figure 3). The Ch3 group most closely corresponds to the horizontal limb of the DBB. It extends from the septal-preoptic region medially to the amygdaloid region laterally. Only 1% of its neurons can definitely be proven to be cholinergic; furthermore, this group is the major source of basal forebrain projections to the olfactory bulb (Figure 4) (Mesulam et al., 1983, 1984).

**Figure 5:**
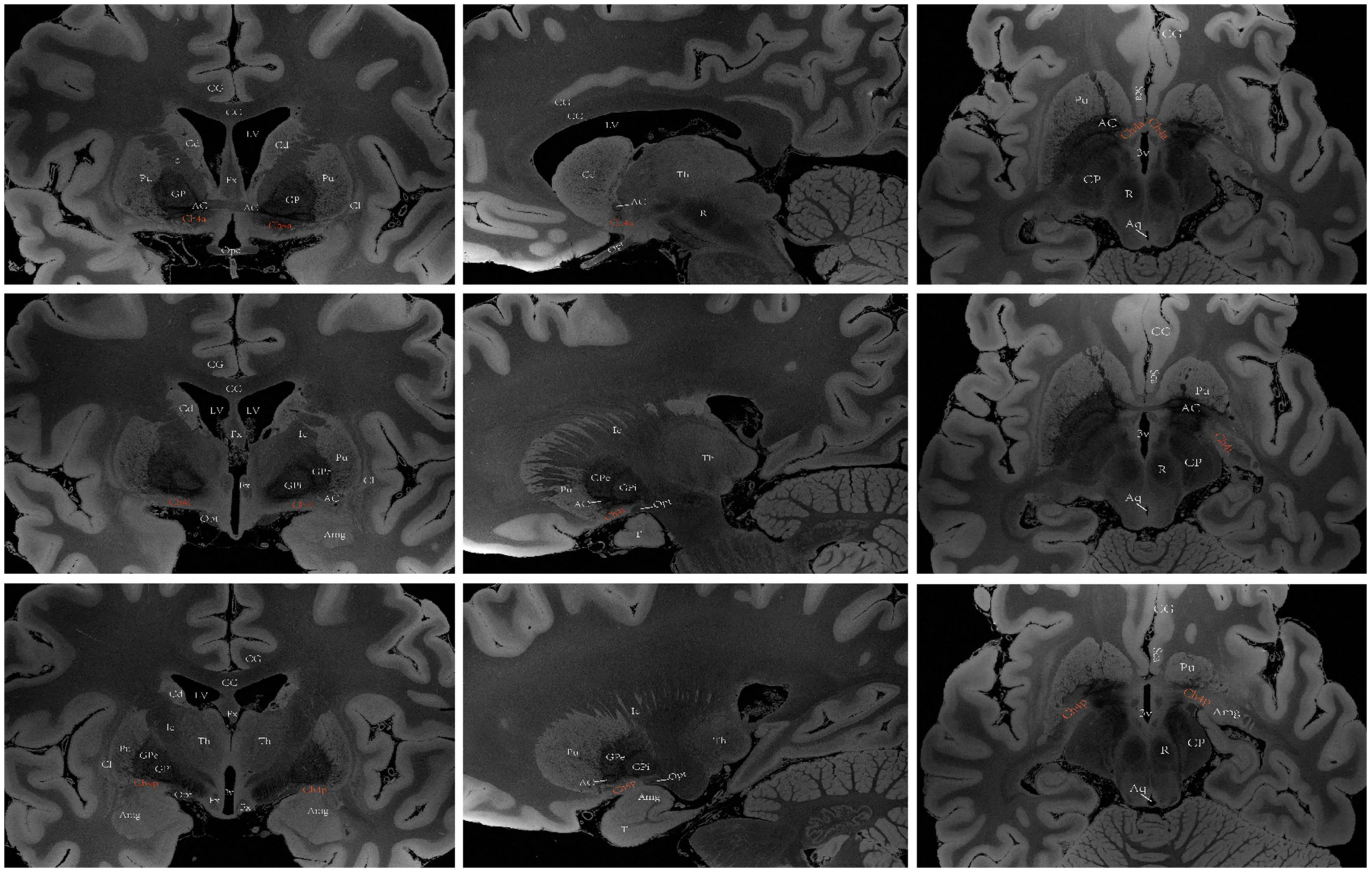
A diagram depicting the location of the human Ch4 on MRI. Images sourced from https://openneuro.org/datasets/ds002179/versions/1.1.0.33 (Marceglia et al., 2021). Coronal (left), sagittal (middle), axial (right). Ch4a (top), Ch4i (middle), Ch4p (bottom). LV lateral ventricle, CG cingulate gyrus, 3 V third ventricle, Fx Fornix, T temporal lobe, Th thalamus, Cd caudatum, Cl claustrum, Gp globus pallidus, GPi internal globus pallidus, GPe external globus pallidus, Pu putamen, Ic Internal capsule, Opt optic tract, Opc optic chiasma, CC corpus callosum, ScA subcallosal area, AC anterior commissure, Amg Amygdala, CP cerebral peduncle, Aq aqueduct of midbrain, R red nucleus.

Ch4 encompasses a complex network of basal nuclear giant cells, including the substantia innominate (SI), NBM, preoptic nucleus, and ventral pallidum. Ch4 is further divided into five subregions: the anterior portion (Ch4a), which is subdivided into anteromedial (Ch4am) and anterolateral (Ch4al) regions; the middle portion (Ch4i), which is subdivided into dorsal middle (Ch4id) and ventral middle (Ch4iv) regions; and the posterior portion (Ch4p). (Mesulam et al., 1983) In humans, the Ch4 nucleus is situated in the posterior region of the basal forebrain and measures approximately 13–14 mm in length and 16–18 mm in width. However, a distinct subregion known as Ch4ai exists between Ch4a and Ch4i within the human brain; this subregion may have emerged due to an increased demand for cholinergic neurons and projection fibers to accommodate the expanded lateral surface area of the neocortex during the evolution of primates into *Homo sapiens* (Mesulam and Geula, 1988). Liu et al. (2015) proposed a conceptual definition for the anterior, intermediate and posterior subsectors of the human Ch4 to simplify its original classification. The anterior division of Ch4 (Ch4a) is situated laterally to the supraoptic nucleus and ventrally to the anterior commissure. The intermediate division (Ch4i) is located in the inferior part of the globus pallidus, medially adjacent to the lateral end of the anterior commissure. At this level, the intermedullary lamina divides the globus pallidus into internal and external components, with the lateral end of the anterior commissure located ventral to the putamen and sometimes the infundibulum visible. The posterior division (Ch4p) is anatomically connected to the globus pallidus medially and superiorly, the putamen laterally and superiorly, and the amygdala inferiorly; furthermore, Ch4p passes through the optic tract medially (Figure 5) (Liu et al., 2015).

Mesulam MM et al. utilized HRP retrograde tracer and acetylcholinesterase (AChE) colocalization technology to effectively demonstrate that cortical topographical innervations originate from distinct subregions of CH4 in the macaque brain. In summary, Ch4a cells innervate limbic regions. Specifically, Ch4am projects to medial cortical regions such as the cingulate cortex, while Ch4al targets fronto-parietal opercular regions and the amygdala. Additionally, Ch4p is responsible for superior temporal and temporal polar regions, while Ch4i covers all remaining cortical areas (Mesulam et al., 1983). By examining human cadaveric brain tissue sections and performing diffusion tensor imaging (DTI) studies, it was discovered that cholinergic efferent fibers originating from the NBM converged into medial and lateral bundles before diffusing throughout the target cortex. The medial bundle fibers converge toward the rostrum of the corpus callosum, penetrate into the cingulate gyrus, and extend posteriorly to reach the splenium and retrosplenial white matter. These axons emanate from the bundle to innervate various regions, including the medial orbitofrontal cortex, subcallosal cortex, cingulate cortex, pericingulate cortex and retrosplenial cortex. The lateral bundle comprises two additional divisions. The capsule division traverses the external capsule and projects fibers to the amygdala and temporal cortex, while the perisylvian division initially enters white matter surrounding the claustrum before radiating laterally to innervate the frontoparietal cortex, superior temporal gyrus, and insula (Selden et al., 1998; Hong and Jang, 2010).

Notably, not all magnocellular neurons in the basal forebrain are cholinergic, and basal forebrain cholinergic neurons are interspersed with noncholinergic neurons and distributed across these nuclei. Therefore, the terms NBM and Ch4 cannot be used interchangeably. Additionally, NBM afferent fibers are primarily derived from the central amygdala, with additional nonsensory and nonmotor cortical input originating from the orbitofrontal cortex, anterior insular lobe, temporal pole, entorhinal cortex, and medial temporal lobe. Subcortical input is received from the diencephalon (midthalamic nucleus and lateral hypothalamus) as well as brainstem structures such as the tegmental area of the midbrain, pontine reticular formation, and nucleus tractus solitarius. These synaptic inputs to the NBM include cholinergic, catecholaminergic, and γ-aminobutyric acid (GABA) axons (Jones et al., 1976; Woolf and Butcher, 1982; Russchen et al., 1985; Kasa, 1986; Mesulam and Geula, 1988; Jones and Cuello, 1989; Smiley and Mesulam, 1999; Zheng et al., 2018). From an anatomical perspective, in addition to emitting cholinergic projection fibers to the neocortex, the NBM also forms bidirectional projections with numerous structures within the limbic system and Papez loops.

### 3.2 Electrophysiology properties of the NBM

Cholinergic neurons in the NBM possess a resting membrane potential of approximately −45 mV and are equipped with voltage-gated ion channels, including fast-activated and rapidly inactivated Na + channels as well as fast-activated and delayed rectification K+ channels (Song et al., 2013; Morelli et al., 2017). Additionally, these neurons are equipped with various ligand-gated ion channels, such as ACh, nicotinic receptors (Morelli et al., 2017), muscarinic receptors (Khateb et al., 1993), epinephrine α1 and β receptors (Fort et al., 1995), histamine H1 and H2 receptors (Khateb et al., 1995; Knoche et al., 2003; Luo and Leung, 2009) and GABA receptors (Khateb et al., 1998; Manfridi et al., 2001). These properties enable the NBM to respond excitably or inhibitably to various neuromodulators, including ACh, nerve growth factor (NGF), estrogen, GABA and dopamine. Cholinergic neurons in the NBM account for approximately 5% of all neurons in this region and exhibit high-frequency pulse bursts that have been shown to be correlated with cortical activation, including cortical γ and θ oscillations; these bursts occur during both awake (AW) and paradoxical sleep (PS) states but are almost completely absent during slow-wave sleep (SWS) (Lee et al., 2005). Spencer JP et al. examined the induction of γoscillations in rat hippocampal slices by AChE inhibitors *in vitro* (Spencer et al., 2010). Chernyshev BV et al. discovered a significant negative correlation between cortical δ oscillation power and the spontaneous firing rate of cholinergic neurons in the NBM by simultaneously recording basal forebrain cholinergic cell firing rates and frontal lobe EEG in spontaneous behavior rabbits; the results indicated that cortical δ oscillation power can serve as an indicator of the activity of cholinergic neurons in the NBM (Chernyshev et al., 2004). These studies demonstrated the indispensable role of ACh pathways in maintaining normal neural network function.

## 4 The role of the NBM in neural network activation and modulation

### 4.1 Evidence from NBM injury animal models

An increasing number of researchers have focused on alterations in neural network oscillations in numerous animal models of NBM injury (Table 1). These findings indicated that NBM lesions caused some consistent changes in neural network oscillations: (1) an increase in the total power of electroencephalography (EEG), particularly in low-frequency bands (δ and θ); (2) a significant decrease in high-frequency oscillations, especially in the γ band; and (3) a reduction in event-related oscillation (ERO) phase-locking index (PLI) (Berntson et al., 2002; Rispoli et al., 2004, 2008, 2013; Sanchez-Alavez et al., 2014; Sanchez-Alavez and Ehlers, 2016). Despite these consistent changes, Rispoli et al. (2013) performed a quantitative EEG (qEEG) analysis of θ oscillations in the hippocampus and the entire EEG spectrum in the cerebral cortexes of rats with bilateral NBM lesions induced by 2-amino-3-(3 hydroxy-5-methylisoxazol-4-yl)-propionic acid (AMPA); the results showed increased power of θ oscillations in the frontal parietal cortex but not in the hippocampus, indicating that the EEG effect of NBM lesions might depend on brain regions. Ljubojevic et al. (2018) reported that the accuracy of visual and olfactory target detection was lower in rats with NBM lesions induced by the selective cholinergic immunotoxin 192 IgG-saporin than in healthy controls; furthermore, the detection accuracy was correlated with decreased local field potential (LFP) coherence in the β range between the prefrontal and posterior parietal cortex and with decreased β power in the posterior parietal cortex before the target’s appearance. Ciric et al. (2016) performed sleep EEG monitoring and found that δ-amplitude attenuation in the sensorimotor cortex during rapid eye movement sleep (REMS) was the earliest indication following bilateral NBM lesions induced by ibotenic acid (IBO) (Ciric et al., 2016). Similarly, Chernyshev et al. (2004) reported a significant negative correlation between cortical δ power and the spontaneous firing rate of cholinergic neurons in the NBM through simultaneous recordings of basal forebrain cholinergic cell firing rates and frontal lobe EEG signals in spontaneous behavior rabbits (Chernyshev et al., 2004). These two studies above indicating that cortical δ oscillation power can serve as an indicator of the activity of cholinergic neurons in the NBM, and future targeted studies are needed to verify this association and explain the underlying mechanisms. Rispoli et al. (2013) examined the use of Huperzine A, a natural selective AChE inhibitor; after this inhibitor was intraperitoneally injected into rats with NBM lesions, the EEG architecture was partially reversed, and desynchronization and reduced θ-power were observed in the frontal and parietal cortexes. Spencer et al. (2010) revealed that the use of AChE inhibitors induced γ oscillations in rat hippocampal slices *in vitro*. These two studies provide further evidence regarding the indispensable role of ACh pathways in the maintenance of normal neural network functioning within the hippocampus.

**Table 1:** Effects of the NBM on the neural network –evidence from NBM injury animal models.

Study	Subjects (n), NBM lesion method	Recording method	Results
Knox and Berntson (2008)	Sprague–Dawley rats (24), the selective cholinergic immunotoxin 192 IgG-saporin	Prefrontal and retrosplenial cortex EEG	During anxiety-like states, increases in the γ power/δ power ratio and augmented θ power were observed on prefrontal and retrosplenial cortical EEGs. NBM lesions attenuated anxiety-like states and the γ power/δ power ratio but had no effect on increased θ power. The effect of NBM lesions on the γ power/δ power ratio was influenced by the effect of increased δ power after lesions.
Ljubojevic et al. (2018)	Long-Evans rats (20), the selective cholinergic immunotoxin 192 IgG-saporin	Prelimbic frontal cortex (PFC) and the posterior parietal cortex (PPC) LFP	Accurate detection of visual and olfactory targets was associated with increased LFP coherence in the β range, between the PFC and PPC, and with increased β power in the PPC before the target’s appearance in sham-lesioned rats. Readiness-associated changes in brain activity and visual and olfactory target detection were attenuated in the NBM lesion group.
Berntson et al. (2002)	Sprague–Dawley rats (19), the selective cholinergic immunotoxin 192 IgG-saporin	Frontoparietal dural surface EEG	Relative to sham-lesioned control animals, the NBM lesion group displayed a significant reduction in high frequency activities on EEG, characterized especially by a reduction in γ power.
Ciric et al. (2016)	Wistar Rats (15), IBO	Sensorimotor cortex EEG	EEG δ amplitude attenuation within sensorimotor cortex during rapid eye movement sleep (REMS) was the earliest sign of aging in the NBM lesion.
Rispoli et al. (2013)	Rats (7), AMPA	Frontoparietal cortex and hippocampus CA1 region EEG	In the NBM lesion group, compared with the control group, an increased θ power in the cortex and a reduced θ rhythm oscillation in the hippocampus were found. In rats with NBM lesions, Hup-A, a selective AChE inhibitor isolated from *Huperzia serrata*, was able to restore EEG architecture, producing cortical desynchronization and reduction in θ power. However, in the hippocampus, this drug increased θ oscillation and reduced the impairment in attention/working memory as well as spatial navigation performance in the behavioral tasks.
Rispoli et al. (2008)	Rats (7), AMPA	Frontoparietal cortex EEG	Bilateral NBM lesions induced an increase of the total EEG power, particularly in δ and θ power, and high-voltage spindle activity the systemic administration of choline pivaloyl esters (CPE) for 3 weeks reversed the above EEG changes.
Rispoli et al. (2004)	Rats (7), AMPA	Frontoparietal cortex EEG	Choline pivaloyl esters (CPE) induced EEG desynchronization, leading to a significant decrease in the total EEG power, especially in the lower frequency δ and θ bands.
Sanchez-Alavez and Ehlers (2016)	Wistar rats (18), AMPA	Frontal cortex EEG and auditory stimuli-ERO	NBM lesions resulted in an increase in auditory stimuli-ERO power in the δ, θ, α and β bands as well as an increase in PLI in the θ band.

n, number of participants or samples; AChE, acetylcholinesterase; LFP, local field potential; IBO, Ibotenic acid; AMPA, 2-Amino-3-(3-hydroxy-5-methylisoxazol-4-yl) propionic acid; EROs, event-related oscillations; PLI, phase locking index; REMS, rapid eye movement sleep.

### 4.2 Neuromodulation effects of NBM-DBS in animal models

Due to the challenges of current drug therapy and the confirmed mechanism of neural network dysfunction in neurodegenerative diseases, there is an urgent need for research on neuromodulation strategies, especially NBM-DBS. Previous animal studies have provided evidence that NBM-DBS could improve cognitive function in AD models, for example, Liu et al. (2018) showed that NBM-DBS could improve performance in monkeys on a continuous performance task, Lee et al. (2016), Nagasaka et al. (2017), and Huang et al. (2019) demonstrated that NBM-DBS could improve spatial memory performance in AD mice, as assessed by Morris water maze. While these animal studies have focused on behavioral outcomes, cellular and molecular mechanisms, and neural network effects. Relatively consistent findings indicate that NBM-DBS leads to decreased power in θ and α oscillations but increased power in γ oscillations in the visual and auditory cortexes and prefrontal cortex (Table 2) (McLin et al., 2002, 2003; Weinberger et al., 2006; Goard and Dan, 2009; Singh et al., 2022). In addition, Dezawa et al. (2021) reported that NBM-DBS facilitated the release of ACh in the primary somatosensory cortex (S1) of rats with NBM lesions and partially reversed the increase in the peak LFP amplitude in S1 after forepaw stimulation. Goard M et al. performed NBM-DBS on healthy rats and recorded the LFP and neuron spike rate in the primary sensory cortex (V1); the findings revealed that (1) NBM-DBS induces notable changes in the power spectrum of the V1 LFP, including increased γ band power and decreased power at low frequencies (<10 Hz), and (2) NBM-DBS leads to prominent decorrelation among neurons and marked improvement in the reliability of neuronal responses to natural scenes by analyzing each single-neuron response to 30 repeats of a natural movie (Goard and Dan, 2009).

**Table 2:** Neuromodulation effects of NBM-DBS in animal models.

Study	Subjects (n), DBS parameters	Recording method	Results
Goard and Dan (2009)	Long-Evans rats (49), NBM-DBS delivered for 500 ms/trial with 100 Hz and pulse width 100 μs	Primary sensory cortex (V1) LFP simultaneous with NBM-DBS	NBM-DBS caused a marked change in the power spectrum of LFP of V1 area, with an increase in power at 10 to 100 Hz (particularly in the γ band, 30 to 50 Hz) and a decrease at low frequencies (<10 Hz). As quantified by the power ratio (LFP power at 10 to 100 Hz divided by that at 1 to 10 Hz), this effect on spontaneous cortical activity lasted for 5 to 10 s.
Singh et al. (2022)	Rhesus monkeys (2), NBM-DBS delivered for 15 s/trial with 80 Hz pulse amplitude 0.2 mA, width 100 μs	Prefrontal cortex LFP after a trial of NBM-DBS	NBM-DBS decreased α power during the delay interval of working memory tasks. No modulation was observed in the γ power in the delay interval of working memory tasks, which has previously been implicated in the maintenance of working memory.
Dezawa et al. (2021)	NBM injury rats (5) with immunotoxin 192 IgG-saporin, NBM-DBS delivered for 500 ms/trial with 100 Hz and pulse amplitude 0.1 mA, width 100 μs	Primary somatosensory cortex (S1) sensory stimuli-ERO after a trial of NBM-DBS and forepaw stimulation	NBM-DBS facilitated ACh release in the S1 subregion and suppressed subsequent sensory stimuli-ERO to forepaw stimulation.
McLin 3rd et al. (2002, 2003)	Sprague–Dawley rats (7), NBM-DBS delivered for 300 ms/trial, 200 trials/day for 14 days, with 100 Hz and pulse amplitude 0.1 mA, width 200 μs	Auditory cortex EEG after 14 days of NBM-DBS	NBM-DBS decreased θ and α power and increases in γ power for several seconds during and after 6 kHz tone presentation.
Weinberger et al. (2006)	Sprague–Dawley rats (11), NBM-DBS delivered for 200 ms/trial, with 100 Hz and pulse width 200 μs, amplitude 47 or 66 μA	Auditory cortex EEG after a trial of NBM-DBS	NBM-DBS produced a reduction of low frequency power (δ, θ, and α) and an increase in high frequency power, β2 and especially γ waves; (2) the moderate level of stimulation (66 μA) produced a longer duration of α power reduction than did the weaker level of stimulation (47 μA).
Schumacher et al. (2021)	Adult cats (11), NBM-DBS delivered for 4 s/trial, with 50 Hz and pulse width 50 μs, amplitude 100 μA	S1 and visual cortex LFP after a trial of NBM-DBS	(1) NBM-DBS shifted LFP activity from high amplitude-low frequency (asleep-like pattern) to low amplitude-high frequency activity (awake-like pattern); (2) NBM-DBS increased cortical NO, which was blocked by systemic NOS inhibition, and the ability of NBM-DBS to induce cortical LFP activation was severely impaired after blocking NOS activity, suggesting that the NBM is involved in the arousal mechanism through the NO pathway.

n, number of participants or samples; NO, nitric oxide; NOS, nitric oxide synthase; V1, primary sensory cortex; S1, primary somatosensory cortex; ACh, acetylcholine; LFP, local field potential; EROs, event-related oscillations.

### 4.3 Evidence of the role of the NBM in neuromodulation from clinical cases

In recent years, many studies have performed qEEG analysis on clinical patients with diseases related to NBM lesions (Table 3). Wan et al. (2019) examined the role of NBM-visual cortex functional connectivity in α reactivity by analyzing simultaneous EEG-multimodal MRI data from cohorts of young and older adults; the results showed that the decrease in α reactivity in older adults was associated with a reduced volume of the fiber bundles connecting the NBM to the visual cortex quantified by leukoaraiosis volume. Similarly, patients with AD (Schumacher et al., 2020) and DLB (Rea et al., 2021) also exhibited decreased α reactivity, which was correlated with a reduced volume of NBM. Furthermore, Schumacher et al. (2021) found that, compared to healthy controls and MCI-AD patients, MCI-DLB patients generally displayed a slower EEG background due to shifts in power from the β and α bands to the preα and θ bands, which was also linked to decreased NBM volume. The evidence above suggests that general sluggishness of EEG signals, an increase in energy at slow frequencies, and a decrease in α reactivity are common characteristics in the process of both natural aging and neurodegenerative diseases and may be associated with lesions of the NBM and its projected fibers. However, the observed decrease in γ oscillation power in animal models with NBM lesions has not been replicated in humans. This may be due to the poor spatial resolution of EEG analysis in humans, which reduces the accuracy of local cortical function represented by γ oscillations compared to LFPs generally applied in animal models.

**Table 3:** Effects of the NBM on the neural network: evidence from clinical cases.

Study	Subjects (n)	Recording method	Results
Wan et al. (2019)	Healthy young adults (21) and older adults (40)	Simultaneous EEG-fMRI	Lesions of the fiber tracts linking NBM and the visual cortex quantified by leukoaraiosis volume were revealed in older adults and found to be associated with reduced α reactivity.
Oswal et al. (2021)	Patients with PDD (6) and patients with DLB (5)	MRI, MEG and NBM-LFP	NBM-cortical structural and functional connectivity were correlated within spectrally segregated networks, including the following: (1) a β band network to supplementary motor area, and activity in which was found to drive the β band activity in the NBM; (2) a δ/θ band network to medial temporal lobe structures encompassing the parahippocampal gyrus and visual areas including lingual gyrus, revealed that NBM networks are likely to subserve in motor control, memory and visual function, respectively.
Schumacher et al. (2020)	Patients with LBD (including 24 patients with DLB and 17 with PDD), AD (21), and healthy controls (40)	EEG and MRI	α reactivity was reduced in AD and LBD patients compared to healthy controls, and a significantly greater reduction was observed in LBD patients than in AD patients. Reduced α reactivity was associated with decreased volumes of the NBM across all groups, especially in the PDD group.
Schumacher et al. (2021)	Patients with MCI-DBL (37), patients with MCI-AD (34), and healthy controls (31)	EEG and MRI	There was a general slowing of the EEG in MCI-DBL patients compared to healthy controls and MCI-AD by a shift in power from β and α bands toward slower frequencies of the preα and θ bands, and a greater reduction in NBM volume was correlated with more severe EEG slowing.
Rea et al. (2021)	Patients with PDD (31), patients with MCI (21), and healthy controls (21)	EEG and MRI	(1) PDD patients showed increased power in the pre-α band (5.5–8 Hz) and reduced α reactivity compared to healthy controls; (2) the volumes of cholinergic cell clusters corresponding to the MS, vertical and horizontal branches of the diagonal band, and the posterior NBM, were positively correlated with α reactivity in patients with PD and pre-α power in patients with MCI.
Nazmuddin et al. (2018)	Patients with PDD (2) who underwent bilateral GPi-DBS with one or more electrodes close to or inside the NBM	EEG and NBM-LFP	PDD patients showed increased δ power in the NBM region and decreased temporal cortical connectivity compared to its proximal structures, including the GPi.
Lee et al. (2019)	Patients with MCI-PDD (5) who underwent bilateral NBM/GPi-DBS	Firing rates	In MCI-PDD patients off medication, the neuronal discharge rates were specific to each area, populated by GPi cells (92.6 ± 46.1 Hz), border cells (34 ± 21 Hz), and NBM cells (13 ± 10 Hz), and during the oddball task, firing rates of NBM cells were decreased (from 2.9 ± 0.9 Hz to 2.0 ± 1.1 Hz).
Cappon et al. (2022)	Patients with PDD (6) and DLB (5) who underwent NBM/GPi-DBS	NBM/GPi-LFP, MEG and EEG	(1) a θ band (2–8 Hz) network linking the NBM/GPi to temporal cortex, and a β band (13–22 Hz) network linking the NBM/GPi to sensorimotor cortex; (2) power of the low β (13–22 Hz) band was significantly higher in the GPi in PDD patients compared to DLB, and coherence in the high β (22–35 Hz) band between the GPi and lateral sensorimotor cortex was significantly higher in DLB patients compared to PDD.
Kuhn et al. (2015)	Patients with mild to moderate AD (6) who underwent NBM-DBS	EEG and PET	A decrease in α power and an increase in θ power after NBM-DBS was observed in only one patient; for all the other patients, no significant changes were observed in EEG frequency power after NBM-DBS.

n, number of patients; LFP, local field potential; PDD, Parkinson’s disease dementia; DLB, dementia with Lewy bodies; LBD, Lewy body dementia (LBD), which includes DLB and PDD; MCI, mild cognitive impairment; GPi, globus pallidus.

With the development of advanced techniques such as fMRI and LFP recording by microelectrodes in NBM-DBS, the NBM neural network can now be explored in greater depth in patients with neurodegenerative diseases. Oswal et al. analyzed the LFP of NBMs with DBS electrodes recorded from PDD (6 patients) and DLB (5 patients) patients treated with NBM-DBS, along with MEG and MRI tractography, to explore the intersection between the NBM neural network and fiber junction (Oswal et al., 2021). First, a β band network strongly related to the projection fiber from the supplementary motor area to the NBM was identified, indicating that the supplementary motor region might be the driver of NBM activity. Then, a δ/θ band network was further identified to be strongly related to the projection fiber from the NBM to the parahippocampal gyrus and lingual gyrus, which might play important roles in memory and visual function. Lee DJ et al. observed distinct neuronal discharge rates in internal globus pallidus (GPi) cells (92.6 ± 46.1 Hz), border cells (34 ± 21 Hz), and NBM cells (13 ± 10 Hz) by performing microelectrode recordings through the GPi and NBM in five PDD patients who received bilateral GPi/NBM-DBS. In addition, the oddball task significantly reduced the discharge rate of NBM cells (from 2.9 ± 0.9 to 2.0 ± 1.1 Hz, *p* < 0.05) (Lee et al., 2019). Nazmuddin M et al. recorded LFP in two PDD patients who underwent GPi-DBS with microelectrodes in the GPi and one or more distal contacts close to or inside the NBM and found that δ (1–4 Hz) oscillations were more prominently present in the NBM region than in its vicinity (Nazmuddin et al., 2018). Considering the negative correlation between δ oscillations and spontaneous discharge of cholinergic neurons in the NBM in an NBM lesion animal model (Chernyshev et al., 2004), the often-ignored δ oscillations can serve as a potential indicator of the specific effects and mechanisms of the NBM on neural network oscillations. In the phase I clinical trial of NBM-DBS for AD patients completed by Kuhn J et al., a positive effect was observed in slowing the decline in ADAS-Cog and increasing cortical glucose metabolism on FDG-PET. However, regarding neural network changes, only one patient exhibited significantly decreased power of α oscillations, and the expected increase in high-frequency oscillation power was not observed. (Kuhn et al., 2015) The possible reason is bias due to the small clinical sample size, while another possible reason that cannot be ignored is the complex relationship between neural network oscillations and different types of cholinergic receptors in the cortex. As shown in the study by Rodriguez R et al., cortical muscarinic receptors can also regulate neural networks (Rodriguez et al., 2004), so the effect of NBM-DBS must be affected simultaneously by the muscarinic and nicotine receptors, and the changes of cortical cholinergic receptor types are different in different neurodegenerative diseases (Graham et al., 2002).

### 4.4 Effects of the NBM in arousal, sleep, and epilepsy

Luo T et al. reversed the frontal EEG inhibition effect of isoflurane by stereotactically injecting histamine into the NBM of anesthetized rats under isoflurane, resulting in a shift from burst inhibition to δ activity, which can be blocked by NBM premicroinjection with an H1 receptor antagonist but not by an H2 receptor antagonist (Luo and Leung, 2009). Similarly, Marino J et al. discovered that cortical nitric oxide (NO) was elevated after basal forebrain electrical stimulation but could be inhibited by systemic nitric oxide synthase (NOS). Additionally, blocking the activity of NOS in the sensory cortex of anesthetized cats significantly weakened the ability of basal forebrain electrical stimulation to induce cortical activation, as evidenced by decreased low-frequency high-amplitude oscillatory activity and increased high-frequency low-amplitude activity (Marino and Cudeiro, 2003). Manfridi’s study on sleep EEG in rats revealed that the activation of GABA_A_ and GABA_B_ receptors in NBM neurons led to increased SWS time and decreased wake time and that only the activation of GABA_A_ receptors led to decreased REMS time (Manfridi et al., 2001). Berdiev et al. (2007) discovered that in the WAG/Rij rat (a model of absence epilepsy), NBM lesions (induced by the selective cholinergic immunotoxin 192 IgG-saporin) increased frontal cortical synchronous activity characterized by the number of spike–wave discharges (SWDs), which were successfully inhibited by the NBM/reticular nucleus of the thalamus (RT)-DBS. Combined with the findings of above studies, NBM neurons are involved in modulating arousal, sleep and epilepsy -related neural networks (Table 4).

**Table 4:** Effects of the NBM on the neural network in arousal, sleep and epilepsy.

Study	Subjects, (n)	Recording method	Results
Luo and Leung (2009)	Long Evans rats (8)	Frontal cortex EEG	Injection of histamine to the NBM shifted the EEG activity from burst suppression pattern by isoflurane toward delta activity, and histamine-evoked activation of EEG was blocked by NBM injection with a H1 receptor antagonist, triprolidine, rather than a H2 receptor antagonist.
Marino and Cudeiro (2003)	Adult cats (11)	Primary somatosensory (S1) and visual cortex LFP after a trial of NBM-DBS	NBM-DBS increased cortical NO, which was blocked by systemic NOS inhibition, and the ability of NBM-DBS to induce cortical LFP activation was severely impaired after blocking NOS activity. These findings suggested that the NBM is involved in the arousal mechanism through the NO pathway.
Manfridi et al. (2001)	Albino rats (13)	EEG and nuchal electromyographic (EMG) monitor states of sleep and wakefulness	Unilateral injection in the NBM with muscimol hydrobromide (a GABA_A_ receptor subtype agonist) and baclofen (a GABA_B_ receptor subtype agonist) induced an increase in slow-wave sleep (SWS) time and an inhibition of wakefulness, while muscimol hydrobromide, but not baclofen, caused a decrease in rapid eye movement sleep (REMS) time.
Berdiev et al. (2007); Berdiev and van Luijtelaar (2009)	WAG/Rij rats (28)	Frontal cortex EEG	NBM lesions induced by injections of 192 IgG-saporin increased spontaneous spike-and-wave discharges (SWDs, a charact of absence epilepsy), while the peaks of the SWDs were less sharp in the hemisphere with the NBM lesion. There was a broader SWD peak frequency in the lesioned hemisphere than in the intact hemisphere (17–18 Hz range vs. 8.3–8.8 Hz), and the injection of carbachol, a cholinergic agonist, in the NBM decreased the number and the mean duration of SWDs.

n, number of participants; S1, primary somatosensory cortex; LFP, local field potential; NO, nitric oxide; NOS, nitric oxide synthase; SWDs, spike-and-wave discharges; SWS, slow-wave sleep; REMS, rapid eye movement sleep.

## 5 Future research prospects of the NBM for neuromodulation

Neuromodulation, represented by DBS and brain-computer interfaces (BCIs), is undoubtedly a promising therapeutic method that meets the urgent need for more effective therapies for a range of debilitating diseases and conditions, including inflammatory and autoimmune disorders, obesity, diabetes, cardiovascular disease, cancer, neurodegenerative and neuromuscular disorders and paralysis (Fetz, 2007; Jackson and Fetz, 2011; Orsborn and Carmena, 2013). The aforementioned findings collectively underscore the pivotal role of the NBM in the neural network, thereby emphasizing the need to investigate its function within the neural network by using advanced techniques such as optogenetics, multielectrode recording, and computer modeling to unravel the underlying mechanisms. Such research will serve as a fundamental basis for precise modulation via DBS and BCIs in the future (Wang, 2010; Bensmaia and Miller, 2014; Shenoy and Carmena, 2014; Guidetti et al., 2021; Marceglia et al., 2021). Moreover, with regard to cholinergic neurons in the NBM that have diffuse projections and diverse functions, it is crucial to avoid treating them as a homogeneous population. Instead, distinguishing them based on histological differences is imperative. Furthermore, cholinergic neurons account for only 5% of the neurons in the NBM; the other cellular components of the NBM have received little attention (Buzsaki et al., 1988; King et al., 1998; Lee et al., 2005). The involvement of other cell types, including glial cells, interneurons, glutamatergic neurons, etc., in each anatomical subdivision unit of the NBM poses an inevitable challenge for investigating the underlying neural network mechanism.

Considering the complexity of neural networks and the multi-site damage of brain tissue in neurodegenerative diseases, it is necessary to break the boundary of single nucleus, and future neural network research needs to be integrated from the perspective of the whole brain. On the one hand, in addition to cholinergic neurons, other neurons or receptor components, such as serotonergic (Viana et al., 2021), GABAergic (Iaccarino et al., 2016), glutamatergic neurons (Xu et al., 2015) and even muscarinic receptor (Velazquez-Moctezuma et al., 1991) also play important roles in the generation and modulation of neural network oscillations. On the other hand, in addition to NBM, other sites, such as the dorsal raphe nucleus (Qamhawi et al., 2015), entorhinal cortex (Leng et al., 2021), and hippocampus (Moodley and Chan, 2014) also play an important role in the development of neurodegenerative diseases, this may explain why so far evidence for NBM-DBS of improving cognitive function in clinical AD patients is rather scant, so dual-target NBM-DBS in the treatment of neurodegenerative diseases is a promising strategy and worth exploring in the future.

## 6 Conclusion

The NBM plays an important and complex role in the neural network dysfunction of various neurodegenerative diseases. The above studies demonstrated that lesions in the NBM can induce alterations in neuronal firing patterns within the prefrontal cortex, auditory cortex, and visual cortex, leading to a reduction in γ oscillation power and an increase in θ and α oscillation power. The underlying mechanism for these changes primarily involves the degradation of cholinergic neurons within the NBM and their cortical projection fibers; however, the contribution of other cellular components within the NBM, such as interneurons, should not be disregarded. Animal experiments utilizing NBM-DBS have shown that NBM-DBS partially reverses neural network abnormalities following NBM lesions, as indicated by decreased cortical θ and α oscillation power, increased γ oscillation power, and improved animal memory function. Although clinical trials investigating the effectiveness of NBM-DBS may not demonstrate outcomes as significant as studies in animal models, delayed cognitive decline associated with AD has been observed. With further advancements in our understanding of neural networks and the development of neuromodulation technologies, neuromodulation based on the NBM will play a transformative role in treating neurodegenerative disorders.

## Author contributions

LJ: Data curation, Software, Writing – original draft. HK: Conceptualization, Data curation, Writing – review & editing. YG: Investigation, Software, Writing – review & editing. XL: Data curation, Formal analysis, Writing – review & editing. MW: Software, Writing – review & editing. KS: Project administration, Resources, Supervision, Writing – review & editing.

## Glossary

**Table:** 

Event-related oscillations (EROs): EROs are a type of brainwave that occur in response to specific events or tasks. They are characterized by regular, rhythmic changes in the brain’s electrical activity, with periods of high frequency (up to 30 Hz) and low amplitude, lasting from a few milliseconds to a few seconds. EROs are typically observed in the alpha, beta, and gamma frequency ranges, and they are thought to play a role in cognitive processing, attention, and working memory and can be measured using various types of EEG technology.
Phase lock index (PLI): The phase lock index is a measure of the synchronicity of the phase angle as a function of frequency and time relative to the stimulation initiation of each trial. The PLI ranges from 0 to 1.0, a higher PLI value of a certain time and frequency indicates that the phase angle of the time and frequency does not change much between tests (Sanchez-Alavez et al., 2014).
α reactivity: α reactivity is the decrease in power of α oscillations recorded in EEG by occipital electrodes after eye opening, and it has been considered to be a potential marker of cholinergic system integrity (Wan et al., 2019).
CFC: Cross-frequency coupling is one form of neural oscillatory coupling that refers to the statistical relationship between a combination of amplitude, phase, and frequency of two distinct frequency bands. There are four types of CFC: amplitude-amplitude coupling (AAC), phase-amplitude coupling (PAC). phase-frequency coupling (PFC) and phase-phase coupling (PPC) (Hanna et al., 2020).
